# How Does Cholecystectomy Influence Recurrence of Idiopathic Acute Pancreatitis?

**DOI:** 10.1007/s11605-016-3269-x

**Published:** 2016-09-23

**Authors:** Claire L. Stevens, Saleh M. Abbas, David A. K. Watters

**Affiliations:** Department of Surgery, University Hospital Geelong, PO Box 281, Geelong, 3220 Australia

**Keywords:** Cholecystectomy, Pancreatitis, Recurrence

## Abstract

**Background:**

Idiopathic acute pancreatitis is diagnosed in approximately 10–30 % of cases of acute pancreatitis. While there is evidence to suggest that the cause in many of these patients is microlithiasis, this fact has not been translated into a resource efficient treatment strategy that is proven to reduce recurrence rates. The aim of this study was to examine the value of prophylactic cholecystectomy following an episode of acute pancreatitis in patients with no history of alcohol abuse and no stones found on ultrasound.

**Methods:**

This was a retrospective study of 2236 patients who presented to a regional Australian hospital. Patients were included when diagnosed with acute pancreatitis with no confirmed cause. Recurrence of acute pancreatitis was compared between those that did and did not undergo cholecystectomy.

**Results:**

One hundred ninety-five consecutive patients met the study definition of “idiopathic” acute pancreatitis. 33.8 % (66/195) underwent cholecystectomy. The patients who had cholecystectomy had a recurrence rate of 19.7 % (13/66) whereas, of those managed expectantly, 42.8 % (68/159) had at least one recurrence of acute pancreatitis (*P* = 0.001).

**Conclusions:**

Following an episode of acute pancreatitis with no identifiable cause, in patients fit for surgery, cholecystectomy should be considered to reduce the risk of recurrent episodes of pancreatitis.

## Introduction

Gallstones and alcohol are by far the most common causes of cases of acute pancreatitis. However, a definite cause is unable to be determined in 10–30 % of patients so that a diagnosis of idiopathic acute pancreatitis is assumed.^1^ The 2013 guidelines published by the American College of Gastroenterology and the IAP/APA Working Group 2 ^,^ 3 suggest that patients with idiopathic acute pancreatitis warrant further assessment by endoscopic ultrasound or magnetic resonance cholangiopancreatography to identify microlithiasis, neoplasms or chronic pancreatitis. However, these recommendations are weak, based on low quality evidence, and access to such investigations is often limited within the public health system.

Microlithiasis is frequently implicated as a cause of “idiopathic” acute pancreatitis (IAP). There is some uncertainty about the possible role of biliary microlithiasis as a causative factor for pancreatitis as it is possible that pancreatitis itself modifies the contents of the gallbladder.^1^^,^ 4 However, treatment of presumed microlithiasis and biliary sludge with cholecystectomy has been shown to prevent subsequent attacks.^4^^,^ 5 While both terms are often used interchangeably, microlithiasis is the presence of stones less than 3 mm in diameter, and biliary sludge is a suspension of cholesterol monohydrate crystals or calcium bilirubinate granules. Previous studies have shown that microlithiasis is present in 70–75 % of IAP patients, and subsequent cholecystectomy or treatment with a cholelitholytic bile acid prevents relapse when microlithiasis is confirmed.^4^^–^ 6 However, more recent long-term follow-up study suggested that microlithiasis is not a significant cause of IAP in a predominantly male population.^7^

The optimal method for detection of microlithiasis or biliary sludge is yet to be determined, and trans-abdominal ultrasound has a significant false negative rate.^1^ When examining liver function tests in inpatients with pancreatitis, an alanine transaminase (ALT) level of greater than 150 IU/L is regarded as the best serum indicator of gallstones as the aetiology.^8^

The definition of idiopathic acute pancreatitis varies between published articles.^1^ The most stringent requires an extensive evaluation that is beyond the scope of most public hospital systems. For the purposes of this study, IAP is acute pancreatitis where a causative factor cannot be determined through history, physical examination, laboratory studies and non-invasive imaging such as trans-abdominal ultrasound or computerised tomography. Where no other cause has been identified and occult cholelithiasis or microlithiasis is suspected and not seen on ultrasound, common practice in some acute surgical units is to perform cholecystectomy at the primary, or more commonly, the second episode of acute pancreatitis. The aim of this study was to examine the value of prophylactic cholecystectomy following recovery from an episode of acute pancreatitis with no stones on ultrasound and no history of alcohol abuse.

## Materials and Methods

This was a retrospective study of 2236 patients that presented to the University Hospital Geelong, Australia with acute pancreatitis from January 2005 through to January 2015. Idiopathic acute pancreatitis was diagnosed where a causative factor could not be determined through history, physical examination, laboratory studies and non-invasive imaging such as trans-abdominal ultrasound or computerised tomography. Patients who had been investigated with trans-abdominal ultrasound, not had prior cholecystectomy, had no identifiable non-biliary cause and had no cholelithiasis seen on ultrasound were included in the study. Non-biliary causes were excluded by close review of clinical notes from hospital admissions, emergency department presentations and outpatient reviews. Radiological investigations were also closely examined. Access to endoscopic ultrasound over the study period was limited, and only four patients underwent this investigation. All four were excluded from the study. To prevent the accidental inclusion of patients affected by alcohol, patients were only included where the clinician had specifically remarked in the notes that alcohol was not thought to be a causative factor. Recurrent episodes of acute pancreatitis were compared between the patients that had been conservatively managed and those that had undergone cholecystectomy.

Data collected included demographics, dates of recurrent episodes or cholecystectomy, serum liver enzyme values, trans-abdominal ultrasound and magnetic resonance cholangiopancreatography (MRCP) results. Where cholecystectomy had been performed, the pathologist’s report findings were recorded.

Recurrent episodes were determined through review of hospital admission records, community, private hospital and public hospital pathology results and radiological studies from three providers within the region at the time of the study.

Statistical analyses were performed using MedCalc for Windows, version 16.1 (MedCalc Software, Ostend, Belgium). Statistical analysis was performed using Fisher’s exact test. *P* values less than 0.050 were considered to be statistically significant. This study was given ethics approval by the Research, Ethics, Governance and Integrity Unit of University Hospital Geelong (Project Number 15/153).

## Results

One hundred ninety-five consecutive patients met the study definition of the study population and were followed for a minimum of 6 and an average of 50 months. One hundred of the patients were male. The median age was 54 years (15–93 years). Of the complete cohort of patients, 73 (37.4 %) had at least one recurrent episode. For all 195 patients, there was another documented medical encounter within the region that confirmed they had not left the region.

### Cholecystectomy Versus Expectant Management

Of the complete cohort of patients, 33.8 % (66/195) underwent cholecystectomy by the end of the follow-up period. The patients who had cholecystectomy had a recurrence rate of 19.7 % (13/66) whereas, of those managed expectantly, 42.8 % (68/159) had at least one recurrence of acute pancreatitis (Table 1). Thirty of the patients that recurred after initial expectant management then went on to have a cholecystectomy after one or multiple subsequent episodes; that decision was made at the discretion of the treating surgeon. Of the 66 patients that underwent cholecystectomy, there was no mortality and one bile duct injury.

**Table 1:** Total patient cohort and rate of recurrence of IAP

Characteristic	Total	Expectant management only (*n* = 129)	Cholecystectomy (*n* = 66)	*P* value
Age, years, median, range	54 (15–93)	55 (15–93)	50 (17–82)	
Gender, male, *n* (%)	100 (51.3 %)	72 (55.8 %)	28 (42.4 %)	0.096
Recurrent episode, *n* (%)		68 (42.8 %)	13 (19.7 %)	0.001

After a primary episode of acute pancreatitis, 159 patients were managed expectantly and 36 had cholecystectomy (Table 2). 42.8 % (68/159) had at least one recurrent episode with expectant management only. A total of 66/195 patients included in the study underwent cholecystectomy, 36 after the primary episode, 23 after a second episode and seven after three or more episodes. Of those that underwent cholecystectomy after their primary episode, 13.9 % had another episode of acute pancreatitis. Of the 68/159 that had a second episode after expectant management (Table 3), 23 underwent cholecystectomy and six of these had a recurrent episode. A similar recurrence rate was seen in the 45 that continued with expectant management after a second episode. Thirteen of these patients had a least one further episode. To enable analysis of the most appropriate time to perform cholecystectomy, the data were analysed separately for a primary episode and second episode (Tables 2 and 3).

**Table 2:** Patient characteristics for patients managed expectantly versus with cholecystectomy after primary episode of idiopathic acute pancreatitis

Characteristic	Total	Expectant management (*n* = 159)	Cholecystectomy (*n* = 36)	*P* value
Age, years, median, range	54 (15–93)	54 (15–93)	54 (24–82)	
Gender, male, *n* (%)	100 (51.3 %)	83 (52.1 %)	17 (47.2 %)	
Recurrent episode, *n* (%)	73 (37.4 %)	68 (42.8 %)	5 (13.9 %)	0.003
Trans-abdominal ultrasound, *n* (%)	195 (100 %)			
Normal	175 (89.7 %)	151 (95.0 %)	24 (67 %)	
Recurrent episode, *n* (%)		61 (40.4 %)	4 (17 %)	0.001
Sludge	14 (7.2 %)	4 (2.5 %)	10 (28 %)	
Recurrent episode, *n* (%)		4 (100 %)	1 (10 %)	0.005
Polyp/s	6 (3.1 %)	4 (2.5 %)	2 (5.6 %)	
Recurrent episode, *n* (%)		3 (75 %)	0	0.143

**Table 3:** Patient characteristics for patients managed expectantly versus with cholecystectomy after a second episode of idiopathic acute pancreatitis

Characteristic	Total	Expectant management (*n* = 45)	Cholecystectomy (*n* = 23)	*P* value
Age, years, median, range	54 (15–93)	54 (15–93)	47 (17–81)	
Gender, male, *n* (%)	33 (48.5 %)	24 (53 %)	9 (39 %)	
Recurrent episode, *n* (%)	19 (27.9 %)	13 (29 %)	6 (26 %)	0.910
Trans-abdominal ultrasound, *n* (%)	68 (100 %)			
Normal	61 (89.7 %)	42 (93.3 %)	18 (78 %)	
Recurrent episode, *n* (%)		11 (26 %)	5 (28 %)	1.000
Sludge	4 (5.9 %)	2 (4 %)	2 (9 %)	
Recurrent episode, *n* (%)		1 (50 %)	1 (50 %)	1.000
Polyp/s	3 (4.4 %)	1 (2 %)	2 (13 %)	
Recurrent episode, *n* (%)		1 (100 %)	0	–

### Radiological Investigations

Trans-abdominal ultrasound showed a normal gallbladder in 175 patients. The number of patients with no ultrasound evidence of stones but either sludge or polyps was 14 and 6, respectively (patients with cholelithiasis or anatomical causes for pancreatitis were excluded from the study). Of those with sludge, 12 eventually underwent cholecystectomy. Expectant management was initially trialled in four of the patients with sludge, but recurrent episodes in two led to cholecystectomy and no subsequent episodes. Ultrasound discovered one or more polyps in six patients, five of which underwent cholecystectomy, with four of those having at least one recurrence prior. No polyposis patients had recurrent pancreatitis after cholecystectomy. One patient of the six had conservative management throughout the follow-up period without recurrence of pancreatitis.

Fifty-one (26.2 %) patients were investigated with MRCP at some time during the follow-up period. In total, 41 patients had a normal result (no cholelithiasis). Twenty-seven patients with a normal MRCP went on with conservative treatment, and 14 underwent cholecystectomy. These patients had five and two recurrent episodes, respectively. A total of 23 patients had an MRCP prior to cholecystectomy. Five of these had sludge, three had tiny stones and one confirmed a gallbladder polyp seen on ultrasound (but was not present on histopathology). None of the patients with cholelithiasis, sludge or microlithiasis seen on MRCP had a recurrent episode of pancreatitis after cholecystectomy. Three patients with small stones on MRCP were excluded from the final analysis.

### Liver Enzymes

In the first 48 h of the primary episode of IAP, the maximum serum value of the liver enzyme ALT was elevated at greater than 150 IU/L in a total of 23 patients. Seventeen went on to have cholecystectomy, and six were managed expectantly with one recurrence in each treatment cohort. Histopathological records of the cholecystectomy specimen reported cholelithiasis in only 13 (19.7 %) of the total 66 cholecystectomy specimens. Ultrasound was normal in eight of these patients, had suggested sludge in three and a polyp in two of the gall bladders. ALT did not predict real cholelithiasis in the patients of this study.

## Discussion

This study represents the first investigation of the value of cholecystectomy for preventing recurrent episodes of acute pancreatitis without the ambiguity and complexity of extensive radiological imaging, genetic testing, investigation of bile composition or invasive endoscopic evaluation. These patients were managed in a real-time hospital environment with standard acute inpatient care; the resources of which included only clinical history and examination, blood tests, trans-abdominal ultrasound and MRCP were obtained if the ultrasound showed no stones. The regional location of the hospital where the study was conducted allowed for excellent long-term surveillance of patients due to the isolated geography and health resources. This study serves as a practical aid to decision-making for the surgeon or gastroenterologist managing acute pancreatitis of uncertain aetiology with readily and realistically available resources.

The most important outcome of our study was to determine that cholecystectomy after the initial episode of acute pancreatitis confirmed with standard investigations including MRCP decreased the risk of a recurrent episode from 43 to 14 % (*P* = 0.003). In addition, recurrent episodes seem to occur with similar frequency regardless of cholecystectomy if it was to be reserved until after a second episode.

The results of this study are supported by a recent randomised controlled trial by Raty et al. 9 that compared watchful waiting with laparoscopic cholecystectomy conducted as an elective procedure. Eighty-five patients were recruited during an outpatient visit after recovery from their episode of pancreatitis and were carefully screened. The outpatient work-up included a thorough focused history, blood tests for alcohol abuse, repeat ultrasonography, genetic testing and MRCP where liver function tests were deranged. Recurrence rates were higher in the control group than the cholecystectomy group (14/46 versus 4/39; *P* = 0.016).^9^

Biliary sludge is almost always an ultrasound diagnosis and was not described until the advent of ultrasound in the 1970s.^10^ Neither the optimal diagnostic method nor the ramification of its diagnosis has been determined with certainty. Biliary sludge is a common finding in patients with acute pancreatitis due to decreased gallbladder distention and stasis. Other situations such as pregnancy, prolonged fasting, total parenteral nutrition, rapid weight loss and post abdominal surgery can also result in reduced gallbladder motility and lead to the development of sludge.^11^ The natural progression for biliary sludge is to disappear, persist or lead to stone formation. The sensitivity of trans-abdominal ultrasound is only 55–60 % for biliary sludge, and for that reason repeat ultrasound, endoscopic ultrasound or bile microscopy has been recommended in cases of IAP.^10^ Given our retrospective study’s significant result in of reduction in recurrence rates following cholecystectomy where sludge is detected on ultrasound, if a patient is suitable for surgery, we believe cholecystectomy is recommended.

When relying on ultrasound (which is what we do in the real world), it is inevitable that patients with and without biliary sludge are going to be included in this group of patients with acute pancreatitis without identifiable cause. Acute pancreatitis without microlithiasis may represent a different group of patients, one which cannot easily be differentiated in clinical practice. As such, the benefits of cholecystectomy in such patients may be due to those patients with sludge or microcalculi. However, we believe the current state of affairs with regards to the imaging supporting decision making favours cholecystectomy during the acute admission for pancreatitis without any other cause.

When deciding whether to offer cholecystectomy during the primary episode of IAP, important considerations in predicting occult biliary disease include the findings of trans-abdominal ultrasound and the predictive value of liver enzymes, in particular alanine aminotransferase (ALT).^8^ This study along with the other available evidence regarding the biliary aetiology of idiopathic acute pancreatitis suggest that one should not wait for patients to have a second episode before performing cholecystectomy. Fit patients without an identifiable non-biliary cause for their IAP should be considered for cholecystectomy. MRCP and repeat ultrasonography have some worth in reassuring both patient and surgeon that the operative course is the correct one, but may delay intervention to until after a further episode of pancreatitis or cloud the possibility of a biliary cause by giving negative result. Certainly, in the unfit or elderly patient, careful consideration should be made to further non-invasive investigation, endoscopic ultrasound, microscopic bile crystal analysis, ERCP with or without sphincterotomy or treatment with a cholelitholytic bile acid.

The limitation of this study is that it has been conducted retrospectively and in a single centre. There was of course an underlying bias in those selected for cholecystectomy that is not readily evident within the limitations of a retrospective audit. The results shown here make clear the need for a prospective study or a possible multicenter randomised control trial looking into the use of only standard inpatient care.

## Conclusion

The results of this study suggest that cholecystectomy is beneficial in preventing recurrent IAP if performed after the primary episode. Further research could seek to determine an accurate selection algorithm to predict which patients would benefit most from cholecystectomy at their primary episode of acute pancreatitis without identifiable cause. However, in patients fit for surgery, prophylactic cholecystectomy should be the default treatment.
